# The management of external marketing communication instruments in health care services

**Published:** 2016

**Authors:** L Bobocea, St Spiridon, L Petrescu, CM Gheorghe, VL Purcarea

**Affiliations:** * “Carol Davila” University of Medicine and Pharmacy, Bucharest, Romania

**Keywords:** external marketing communication, health care services, BCG Matrix, SWOT analysis, Gantt Diagram

## Abstract

In order to become known and attract consumers, a health care organization has to develop suitable external communication campaigns. Consequently, management instruments are employed to effectively evaluate the success of a campaign. The BCG Matrix, SWOT analysis and the Gantt Diagram were used in this paper to ensure the consistency and accuracy of the external communication process at an empirical level.

**1. Management marketing characteristics and management marketing functions**

Management marketing consists of a wide range of activities, such as analysis, planning, implementation, and control, which mainly focus on the creation, promotion, and outcome assessment, to satisfy the desired objectives of a health care organization [**1**].

In the new context of globalization, the health care management marketing of services has the following functions [**2**]:

• The identification of key factors which uncover the success of an organization, as for instance, innovation, financial power, quality of services, qualification of work force;

• The constant monitorization of the competition’s positioning strategy;

• The acknowledgment of the competition’s history, tradition and managerial personalities;

• The identification of the competition’s marketing actions in terms of visibility. 

Still, the classic management functions as described by the scientific literature can also be added to the above-mentioned functions [**3**]:

- The planning function of health care organization’s activities in terms of time and space, objectives and priorities;

- The organizing function;

- The leadership function is reflected in the communication between departments, employees, managers and employees, organizations and other health care providers and health care organizations and consumers, respectively;

- The control function concentrates on the employee supervision and the process of employee analysis;

- The implementation function. 

Briefly, marketing should be used as a complement of the management activities. 

**2. The external marketing communication in health care services**

The health care communication strategy of an organization with its marketing environment is an important condition in surviving and assessing the desired outcomes. An efficient communication is reflected in the development of a system of relations, which encompass the consumer expectations and the long-term interests of the medical unit. 

In most cases, communication has the following main objectives:

- To attract potential consumers using the concepts of positioning and offer;

- To inform the potential consumers about the health care service offers;

- To convince the potential consumers regarding the necessity of buying the health care services.

Currently, it was concluded that communication contributes significantly to the management of buying process, covering not only the pre-buying phase but also the buying phase *per se *[**9**].

Marketing Communication has the role of informing, convincing, influencing and reminding [**4**]. Further, the communication process becomes promotion in the marketing environment. The promotion policy aims to disseminate information about the activities and services of the health care organization at an extensive level, as well as monitor the perceptions of the targeted populations. 

Nonetheless, when a health care organization wants to communicate with its consumers, this may happen on two strategic directions [**9**]:

- Externally, reflected in the external marketing communications;

- Internally, with the help of employees, during the service delivery, meaning the interactive communication. 

A third type of communication also refers to the internal communication but occurs between managers and employees. As such, internal communication is performed between departments or depending on the hierarchic position in the organization. 

The health care units use the external marketing communication instruments to stimulate the targeted population in developing and building a favorable image on the market. This mix of instruments is known in the scientific literature as the promotional communication mix. 

The main objectives of the external communication strategies are the following [**2**]:

- The reputation growth of the health care organization;

- The credibility growth of the health care organization;

- Building a favorable image of the health care organization in the minds of the consumers;

- Triggering a positive buying behavior regarding the services of the health care organization. 

The basic instruments of external communication for sending messages are the following [**2**]:

- *Advertising*

In its turn, advertising uses the following tools: 

- television;

- radio;

- magazines;

- newspapapers;

- banners;

- catalogues, brochures, flyers;

- outside signalization banners. 

* *Sale forces*

The sale force of a health care organization consists of the employees who deliver medical services and other requested information to consumers, partners, and competitive units.

* *Sale promotion*

The sale promotion takes place when free promotional objects such as calendars, address books, pens, and even free consultations are being offered, with the purpose of growing the reputation of the health care organization.

* *Public Relations*

Public Relations are direct contacts with different categories of public on different themes, which occur during congresses, symposiums, workshops, interviews, and press releases. 

* *The Internet*

The instruments used by the internet are online press releases, websites, communication through Social Media platforms, link exchange partnership contacts, the online profile of organizations, discussions on forums. 

* Fairs and exhibitions on health care topics 

* Word-of-mouth is the recommendation of health care services among patients by other patients or third parties.

* Other promotional tools:

- Name, logo and slogan;

- The building’s architecture;

- The inside ambiance;

- The furniture and equipment ergonomics;

- The colors found inside the building and on the logo;

- The uniform of the employees;

- The vehicles.

**3. The efficiency growth of external marketing communication through management instruments**

The message, which uses different external communication channels, is delivered with the help of campaigns. Over time, marketing specialists struggled to measure the efficiency of communication campaigns, but they have concluded that the evaluation process is constantly changing. Subsequently, the investigation of the campaign elements are measured accurately and accordingly as long as it is performed with the help of management instruments [**5**]. 

In Romania, it was acknowledged that the degree of usage of measurement instruments from management is rather scarce when employed in the external marketing communication. As such, 50% of the Romanian organizations use the SWOT analysis, 7% use the Boston Consulting Group (the BCG Matrix) and 17% declared they have no scientific method for the analysis [**6**]. 

In the absence of scientific methods, the Romanian organizations have higher risks of coming across many issues, as for example [**7**]:

- The vulnerability growth in a dynamic and not so well researched environment;

- The market opportunity loss;

- The market share loss in favor of more informed and offensive competitors;

- Ineffective and insufficient promotional costs;

- Unjustified prices of medical services;

- Inter-functional conflict growths.

Even if there is reluctance in employing the management instruments to measure the efficiency of external marketing communication at an empirical level, the implementation process of campaigns is a suitable supportive environment for their application. Therefore, the following instruments offer the possibility to measure the efficiency of an external communication campaign in a health care organization [**8**]: 

1. Boston Consulting Group Model (BCG)

The BCG model is fundamentally built on the relative market share and market growth rate matrix. Each communication activity of a health care organization is represented in a circle-like figure. The circle area is in accordance with the Return on investment value of the marketing communication activity, the coordinates of the circle are given by the relative market share of the health care organization, and the market growth rate is illustrated in contrast to the competition’s. The matrix anticipates a natural evolution of a campaign. Comparing the actual matrix with the forecasted one, experts may identify the major strategic issues during the implementation phase of the campaign. Therefore, four strategies can be employed as it follows (**Fig. 1**): 

- The investment strategy. This strategy seeks to improve the market share on the expense of short-term profit. It is specific to Dilemmas, as they become Stars. 

- The maintenance strategy. This type of strategy seeks to maintain the position gained on the market. It applies to Cash Cows but only in the case of a higher impact on the financial resources of the organization. 

- The fructification strategy. This strategy seeks to raise the financial contribution in a short period of time. It applies to Cash cows, which have an uncertain future. In addition, it may also be applied to Dilemmas and to Dogs. 

- The desertion strategy. This strategy seeks to sell some parts of the organization that have negative effects on the available resources. It applies to Dogs and Dilemmas when the organization is reluctant to invest in them. 

**Fig. 1:**
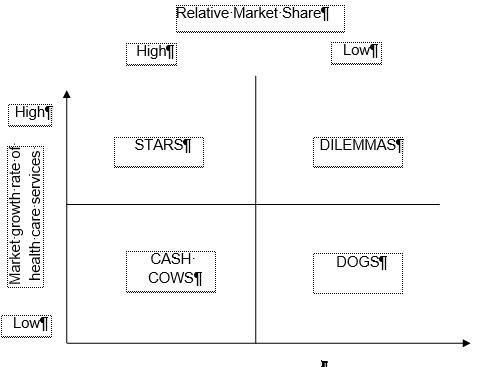
Boston Consulting Group Matrix

2. SWOT Analysis examines the strengths and weaknesses of a health care organization which may bring advantages or disadvantages to an external marketing communication, as well as opportunities and threats found in the external environment of the organization (**Table 1**).

**Table 1:** SWOT Matrix of an external communication campaign in a health care organization

SWOT ANALYSIS	
THE ORGANIZATION (THE INSIDE ENVIRONMENT)	
Strengths	Weaknesses
- Motivated managerial team	- Insufficient human resources
	- Inadequate structure
Opportunities	Threats
- Lack of competition	- The competition with other sectors for budget resources
	- Unsatisfied medical needs
THE OUTSIDE ENVIRONMENT	

3. GANTT Diagram is a time instrument of communication planning of activities. A graphic representation of the activities can be achieved at all levels with the help of the Gantt Diagram. More than one communication activity can be illustrated on the same time frame (**Table 2**).

**Table 2:** Example of Gantt Diagram

No.	Activity	Week 1	Week 2	Week 3	Week 4	Week 5	Week 6
1.							
2.							

4. The limits of the instruments [**8**]

- They give a sense of absolute rationality and infallibility, but reality is not that predictable;

- The instruments are not always suitable for any moment in time;

- The world is a dynamic place, so development and change occurs. The instruments offer a slight prediction in which weaknesses appear in the delivery process. 
